# An analytical stochastic stage-structured model for pest population dynamics: a case study on *Cydia pomonella* in Italy

**DOI:** 10.1007/s00285-026-02461-8

**Published:** 2026-09-16

**Authors:** Berk Tan Perçin, Serena Baiocco, Federico Cavina, Gianfranco Pradolesi, Sara Pasquali

**Affiliations:** 1 CNR-IMATI “E. Magenes”, Via A. Corti, 12, 20133 Milan, Italy; 2 Terremerse Soc. Coop., Via Cà del Vento, 21, 48012 Bagnacavallo, RA Italy

**Keywords:** 92D25, 60G55, 92D45

## Abstract

In this work, we propose a novel phenological model to describe population dynamics based on a compound Poisson process driven development. The model offers an alternative to other well-established approaches founded on systems of ordinary or partial differential equations, in which, the stochasticity is driven by the Brownian motion. A key advantage of the proposed framework is that it prevents the age regression of individuals and allows for the analytical derivation of the number of individuals in each developmental stage into which the population is structured. The model is applied to *Cydia pomonella*, a major pest of pome fruit crops. The results obtained are highly promising and suggest that this approach constitutes a valuable alternative to traditional models based on partial differential equations for simulating the population dynamics.

## Introduction

Understanding the population dynamics of invasive insect pests is essential for the development of effective monitoring and control strategies. Population dynamics are strongly influenced by environmental drivers, particularly temperature, which directly affects species biology and life-history traits. A common modeling approach relies on physiologically based models (Gutierrez 1996), in which each individual is characterized by a physiological age representing its level of development.

Because individuals are dangerous in a particular stage of their life, stage-structured models (Cushing 1998; Iannelli 1995; Iannelli et al. 2017) provide a natural and biologically meaningful framework. In such models, the population is partitioned into developmental stages, each described by stage-specific bio-demographic functions governing development, mortality, and, for adults, fecundity.

Stage-structured physiologically based models (Metz and Diekmann 2014) explicitly describe the response of a species to environmental conditions. Depending on their objectives, these models can be broadly classified as phenological or demographic. Phenological models predict the timing of developmental events by describing the cumulative progression of individuals through successive life stages, whereas demographic models quantify the temporal evolution of stage-specific population abundances. In their simplest formulation, phenological models require only stage-specific development functions, although more comprehensive formulations may also incorporate mortality and fecundity processes (Pasquali et al. 2019). Phenological models are among the most widely used tools in pest management decision support systems because many management strategies primarily depend on the timing of susceptible life stages rather than on the exact population size. By predicting the cumulative proportion of individuals completing each developmental stage, these models provide a reliable basis for scheduling interventions such as pesticide applications or the release of biological control agents. In contrast, demographic models explicitly estimate the abundance of individuals within each life stage over time, thereby describing the dynamics of the entire stage-structured population. Although demographic models provide more detailed information, they generally require additional biological data and a more complex model structure. Consequently, the relatively modest data requirements and straightforward implementation of phenological models have contributed to their widespread adoption in operational pest management decision support systems.

From a mathematical perspective, physiologically based models are commonly formulated either as systems of ordinary differential equations (ODEs) (Blum et al. 2018; Ewing et al. 2016; Marini et al. 2016) or as systems of partial differential equations (PDEs) (Ainseba et al. 2011; Gilioli et al. 2016, 2017, 2014; Pasquali et al. 2020; Sperandio et al. 2024). In the PDE framework, deterministic formulations are typically based on von Foerster-type equations (VanSickle 1977; Rossini et al. 2020), whereas stochastic formulations rely on Kolmogorov equations (Buffoni and Pasquali 2007; Pasquali et al. 2022).

The main advantage of stochastic approaches lies in their ability to account for inter-individual variability in developmental dynamics. In classical formulations, the physiological age satisfies a stochastic differential equation (SDE) driven by a Wiener process (Pasquali 2021). However, this choice may lead to biologically unrealistic regressions in physiological age. To address this issue, Pasquali (2021); Pasquali and Trivellato (2023) proposed modeling physiological age through an SDE driven by a Gamma process, leading to a system of generalized Kolmogorov equations (Eliazar and Klafter 2003; Dubkov and Spagnolo 2005; Dubkov et al. 2008; Denisov et al. 2009).

Both classical and generalized Kolmogorov systems do not admit closed-form solutions and typically require numerical discretization to approximate the density of individuals within each stage, resulting in significant computational effort.

In this work, we introduce a novel stochastic stage-structured phenological model that allows the stage-specific population densities to be derived in closed form. We define the physiological age as the solution of an SDE driven by a compound Poisson process. Under this formulation, the percentage of individuals in each stage can be obtained directly from the probabilistic structure of the SDE, without the need for a system of PDEs. This yields explicit expressions for stage densities instead of resorting to computational simulations for the population dynamics.

The paper is organized as follows. In Sect. 2, we review population dynamics models based on systems of PDEs that are well established in the literature. Section 3 introduces the proposed compound Poisson stage-structured model. In Sect. 4, the new model is calibrated to describe the dynamics of the pest *Cydia pomonella* using the data collected from several fields across Italy. Its performance is then compared with that of the well-established Gaussian model in terms of its ability to capture the pest dynamics observed in the same fields in Italy. Finally, Sect. 5 presents the concluding remarks.

## Population dynamics based on a PDE system

In this section, we review the principal continuous-time modeling frameworks based on PDEs that have been proposed in the literature to describe the dynamics of stage-structured populations. We consider both deterministic and stochastic formulations, presenting the governing equations that characterize the population dynamics in each case.

### Von Foerster equations

Among deterministic approaches, the most widely used framework is provided by the von Foerster equations (VFEs), see (VanSickle 1977). In this work, we consider the formulation without mortality, as our focus is on phenological models that conserve the total number of individuals over time.

Let *s* denote the total number of stages in the population, where stage 1 corresponds to eggs and stage *s* to the reproductive adult stage. Let x∈[0,1] represent the physiological age, defined as the percentage of development within a given stage. Under these assumptions, the von Foerster model describing the stage-structured population dynamics is formulated as the following system of partial differential equations:

2.1 $$\begin{aligned} \begin{aligned} \frac{\partial \phi _i(t,x)}{\partial t} + \frac{\partial }{\partial x}\nu _i(t)\phi _i(t,x)&= 0,\quad t>0,\,x\in (0,1],\\ \left[ \nu _i(t)\phi _i(t,x) \right] _{x=0}&=F_i(t),\\ \phi _i(0,x)&= \hat{\phi }_i(x), \end{aligned} \end{aligned}$$

for $i\in \{1,2,\dots ,s\}$, where $\phi _i(t,x)$ denotes the density distribution of individuals in the *i*-th stage at time *t* with respect to the physiological age variable *x*. The function $\nu _i(t)$ represents the stage-specific development rate, while $\hat{\phi }_i(x)$ denotes the initial age distribution. Finally, $F_i(t)$ is the influx of individuals entering stage *i* at physological age x=0. It is explicitly given by:

2.2 $$\begin{aligned} F_i(t):={\left\{ \begin{array}{ll} \nu _s(t)\int _0^1\rho (x)\phi _s(t,x)dx,& i=1,\\ \nu _{i-1}(t)\phi _{i-1}(t,1),& i\in \{2,\dots ,s\}, \end{array}\right. } \end{aligned}$$

where ρ(x) denotes the oviposition profile of the pest species, i.e., the probability density of egg laying as a function of the physiological age *x*.

Moreover $\int _{0}^{1}\phi _i(t,x)dx$ represents the total number of individuals in stage *i* at time *t*. Together with the development rate, the first term in Eq. (2.2) represents the total number of eggs laid by adults at time *t*. In biological terms, we assume that individuals in the final (reproductive) stage generate new individuals entering the first stage at a rate proportional to their development rate. This assumption ensures the conservation of the total population across successive generations.

For the other stages, individuals that complete their development in stage i-1 transition to stage *i* entering the new stage at physiological age x=0.

### Kolmogorov equations

A major limitation of the von Foerster model is its deterministic nature, which implies that all individuals within stage *i* develop at the same rate $\nu _i$. However, it is well established that development within a given stage exhibits substantial inter-individual variability.

A natural way to account for such variability is to model the physiological age as a stochastic process governed by a stochastic differential equation (SDE). Specifically, for each stage $i\in \{1,\dots ,s\}$ the physiological age is assumed to satisfy:

2.3 $$\begin{aligned} dX^i_t = \nu _i(t)dt + \sqrt{2\sigma _i}dB^i_t,\quad X^i_0=x^i_0\in (0,1), \end{aligned}$$

where $\sigma _i>0$ is a diffusion coefficient, $B^i_t$ denotes a standard Brownian motion, and the Brownian motions associated with different stages are assumed to be independent, i.e., $B^i_t\perp B^j_t$ for i≠j. Under assumption (2.3), the density function of physiological age satisfies the following partial differential equation:

2.4 $$\begin{aligned} \begin{aligned} \frac{\partial \phi _i(t,x)}{\partial t} + \frac{\partial }{\partial x}\left[ \nu _i(t)\phi _i(t,x) - \sigma _i\frac{\partial \phi _i(t,x)}{\partial x} \right]&= 0,\quad t>0,\,x\in (0,1),\\ \left[ \nu _i(t)\phi _i(t,x) - \sigma _i\frac{\partial \phi _i(t,x)}{\partial x} \right] _{x=0}&=F_i(t),\\ \left[ \sigma _i\frac{\partial \phi _i(t,x)}{\partial x} \right] _{x=1}&=0,\\ \phi _i(0,x)&= \hat{\phi }_i(x), \end{aligned} \end{aligned}$$

where $\hat{\phi }_i(x)$ is the initial distribution in stage *i*, and the boundary condition $F_i(t)$ is defined in (2.2).

System (2.4) also represents a phenological model that preserves the total number of individuals in the population. In the limiting case $\sigma _i=0 \; \forall i$, the stochastic contribution vanishes and the model (2.4) reduces to the von Foerster system (2.1). Hence the Kolmogorov model can be seen as a generalization of the von Foerster model.

The main limitation of this stochastic formulation is that the Brownian diffusion permits regressions in physiological age due to Brownian increments taking negative values. Such behavior is biologically unrealistic when physiological age represents a non-decreasing quantity, such as the proportion of development completed within a stage. This issue can be addressed by modeling the physiological age through an SDE driven by a Gamma process, as proposed in Pasquali and Trivellato (2023), leading to a system of generalized Kolmogorov equations. Then, this formulation yields population dynamics analogous to those of the classical Kolmogorov system. When the stage-specific development rates are calibrated properly, so that the expected physiological age under the Gamma-driven model coincides with that of the Wiener-driven model, one obtains comparable results with the ones in the literature. However in this setup, because the system of generalized Kolmogorov PDEs is again a coupled system of PDEs, it is not feasible to approach the system analytically. Instead, one has to discretise and solve the system numerically.

## Introduction of the compound Poisson model

In this section, we introduce a novel modeling framework that not only prevents regressions in physiological age but also allows the population sizes to be derived in closed form, without resorting to a system of PDEs. The model is based on the use of a compound Poisson process to account for variability in physiological age.

### Preliminaries and the logic of the model

Similarly to the Gaussian model (2.3), we suppose that the physiological age $X_t$ of a pest satisfies the SDE:

3.1 $$\begin{aligned} dX_t := \nu (t) dt + dZ_t,\quad X_0 = x_0 \end{aligned}$$

where ν(t) is the deterministic development rate and $Z_t$ is the compound Poisson process, namely:

3.2 $$\begin{aligned} Z_t := \sum _{k=0}^{P_t}G_k, \end{aligned}$$

where $G_k$ are non-negative independent random variables with identical cumulative distribution function (cdf) *F* and $P_t$ is the regular Poisson process defined in Definition 3.1, which is also independent of $G_k$’s for all $k\in \mathbb {N}$.

#### Definition 3.1

A Poisson process with parameter λ>0 is a continuous time stochastic process $\{P_t\}_{t\ge 0}$ such that

1. $\mathbb {P}(P_0 = 0)=1$. In other words, the process starts from 0 a.s.
2. For any 0≤s<t, $P_t-P_s$ is a Poisson random variable with mean λ(t-s), that is
  $$\begin{aligned} \mathbb {P}(P_t-P_s = k)=\mathbb {P}(P_{t-s}=k)=e^{-\lambda (t-s)}\frac{(\lambda (t-s))^k}{k!}, \quad for \, k\in \mathbb {N}. \end{aligned}$$
3. For any $0\le t_1<t_2<\dots <t_n$, the random variables
  $$\begin{aligned} P_{t_1}, \, P_{t_2}-P_{t_1}, \ldots ,\, P_{t_n}- P_{t_{n-1}} \end{aligned}$$
  are mutually independent.
4. The process $P_t$ is almost surely right continuous with finite left hand limits.

The solution to Eq. (3.1) can be written as

3.3 $$\begin{aligned} X_t = x_0 + \int _{0}^{t}\nu (s)ds + Z_t. \end{aligned}$$

Observe that in this model, for fixed t≥0, we perceive $X_t$ to be a non-negative random variable, that will take into account the *age* distribution of individuals at time *t*. Therefore, in this setup the physiological age no longer lies within the interval [0, 1] for a given stage, as in the PDE models presented in Sect. 2. Instead, physiological age will be interpreted as a measure of biological age that reflects structural development rather than chronological time. The concept will be explained in detail below in the logic of the compound Poisson model.

The logic of this model is presented in the box below:

**Figure:**
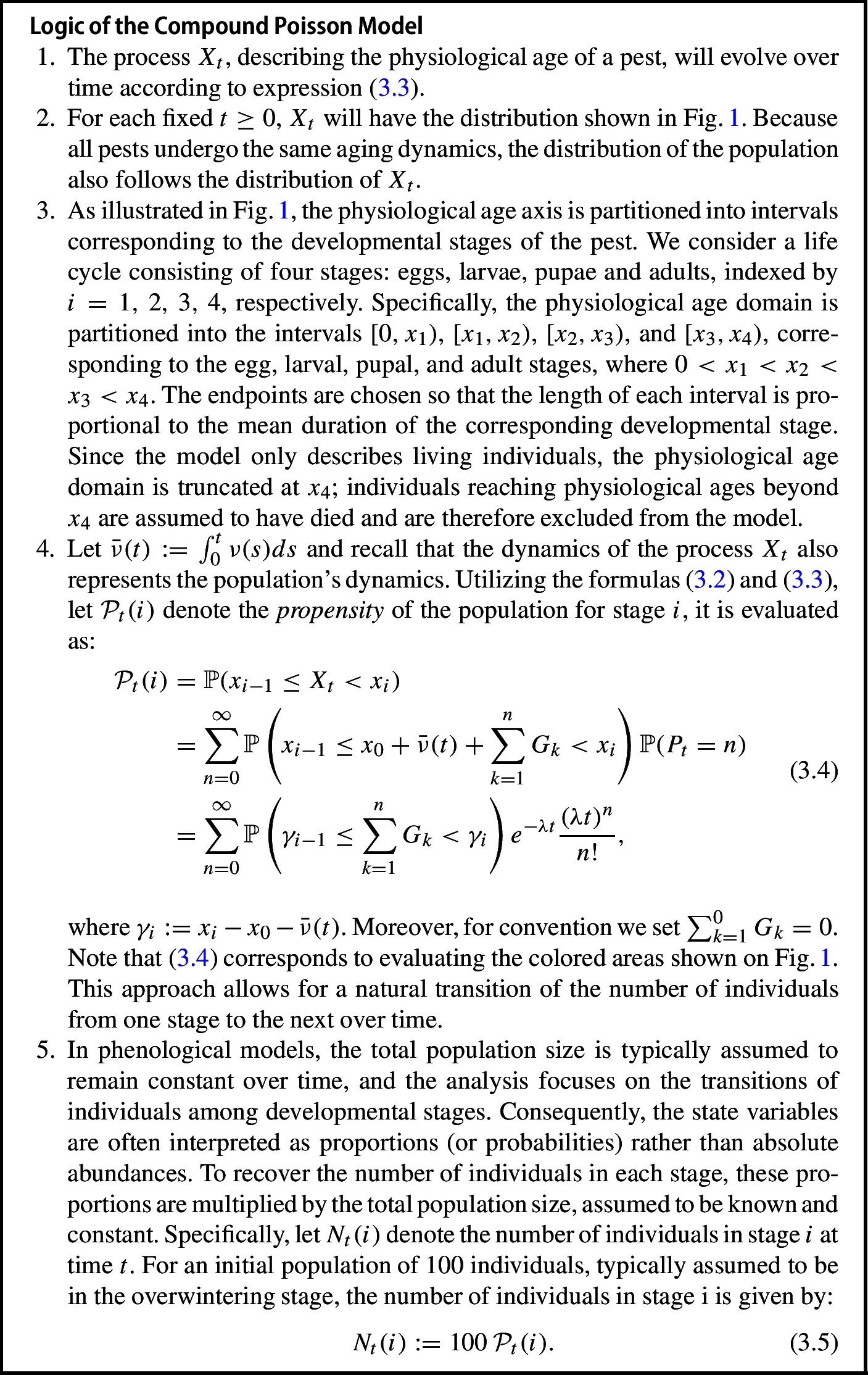

**Fig. 1:**
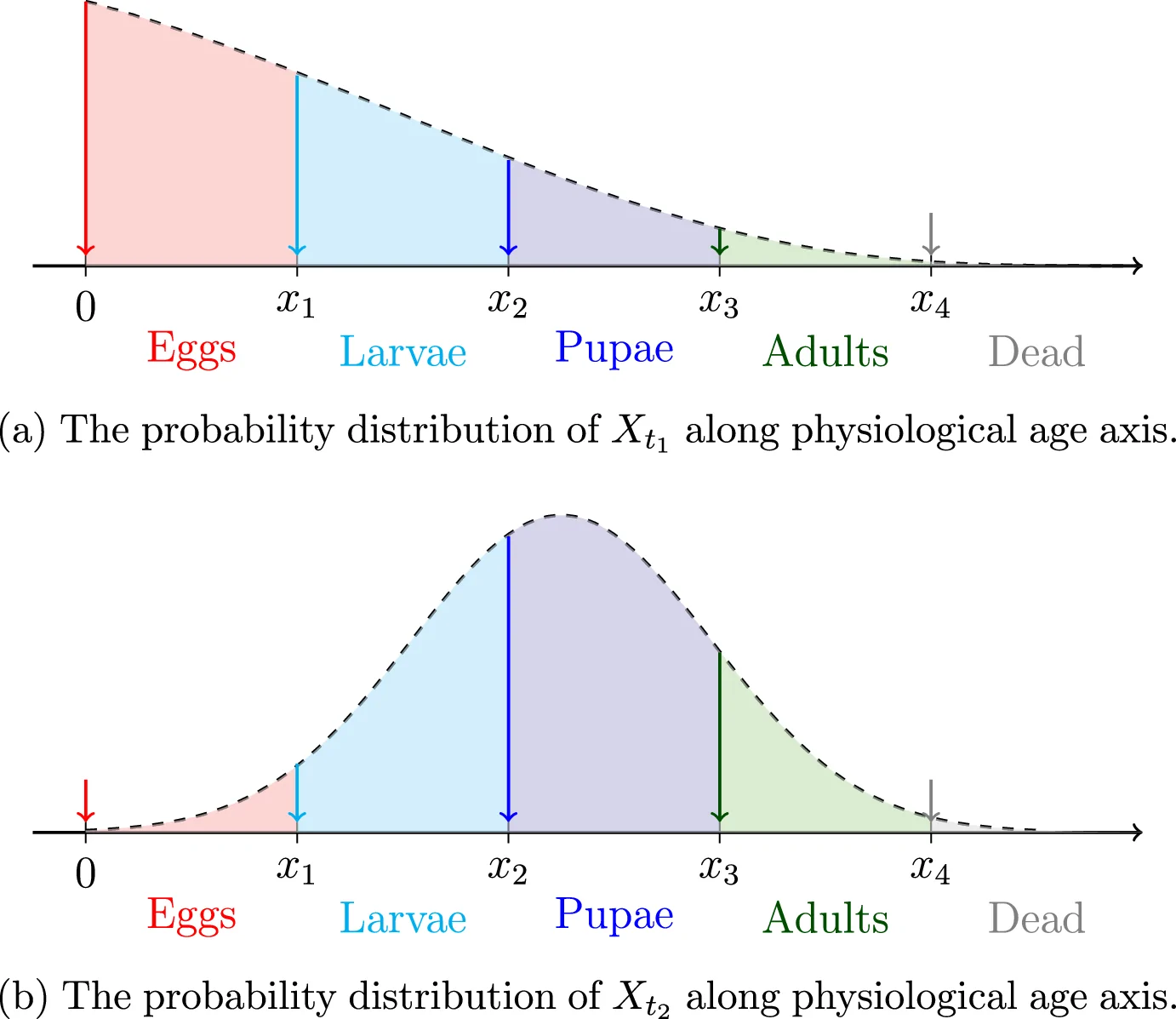
The figure summarizing the new model’s logic. Note how the probability density shifts to later developmental stages with time $t_2 > t_1$

#### Remark 3.1

In principle, it is possible to choose $G_k$ such that they satisfy any distribution and evaluate how the sum is distributed by means of convolution. However, if one chooses the distribution of $G_k$ for any $k\in \mathbb {N}$, as uniform, exponential or chi-squared cdf, then the term $\sum _{k=1}^nG_k$ is explicit. For the purposes of this paper, we choose each $G_k\sim \mathcal {E}xp(\theta )$ with θ>0 as the scale parameter. This choice leads to $\sum _{k=1}^nG_k\sim Erlang(n,\theta )$ for n≥1 making the expression () fully explicit:

3.6 $$\begin{aligned} \mathcal {P}_t(Eggs)&= \sum _{n=0}^{\infty } F_{n,\theta }(\gamma _{1}) e^{-\lambda t}\frac{(\lambda t)^n}{n!}\nonumber \\ \mathcal {P}_t(Larvae)&= \sum _{n=0}^{\infty } \Big ( F_{n,\theta }(\gamma _{2}) - F_{n,\theta }(\gamma _{1})\Big ) e^{-\lambda t}\frac{(\lambda t)^n}{n!}\nonumber \\ \mathcal {P}_t(Pupae)&= \sum _{n=0}^{\infty } \Big ( F_{n,\theta }(\gamma _{3}) - F_{n,\theta }(\gamma _{2})\Big ) e^{-\lambda t}\frac{(\lambda t)^n}{n!}\nonumber \\ \mathcal {P}_t(Adults)&= \sum _{n=0}^{\infty } \Big ( F_{n,\theta }(\gamma _{4}) - F_{n,\theta }(\gamma _{3})\Big ) e^{-\lambda t}\frac{(\lambda t)^n}{n!}.\nonumber \\ \mathcal {P}_t(Dead)&= \sum _{n=0}^{\infty } \Big ( F_{n,\theta }(\gamma _{5}) - F_{n,\theta }(\gamma _{4})\Big ) e^{-\lambda t}\frac{(\lambda t)^n}{n!}, \end{aligned}$$

where

3.7 $$\begin{aligned} F_{n,\theta }(x):=1-\sum _{k=0}^{n-1}\frac{1}{k!}\left( \frac{x}{\theta }\right) ^k e^{-\frac{x}{\theta }}, \end{aligned}$$

is the cdf of an Erlang random variable with the shape parameter $n\in \mathbb {Z}^+$ and scale parameter θ>0.

#### Remark 3.2

Note that this segmentation can be carried out without imposing boundary conditions on the SDE (3.1), since the process evolves only in the forward direction. This follows from the fact that *F* in (3.2) is chosen as the cumulative distribution function of a non-negative random variable. In other words, individuals cannot become younger over time, which represents the major drawback of the Gaussian-driven Kolmogorov model (2.4).

Consequently, there is no need to introduce boundary conditions or to reformulate the problem in the PDE framework starting from the SDE. Instead, the compound Poisson–driven equation can be solved directly, yielding the explicit expression (3.6). This constitutes the main advantage of the compound Poisson model over classical/generalized Kolmogorov PDEs.

**Fig. 2:**
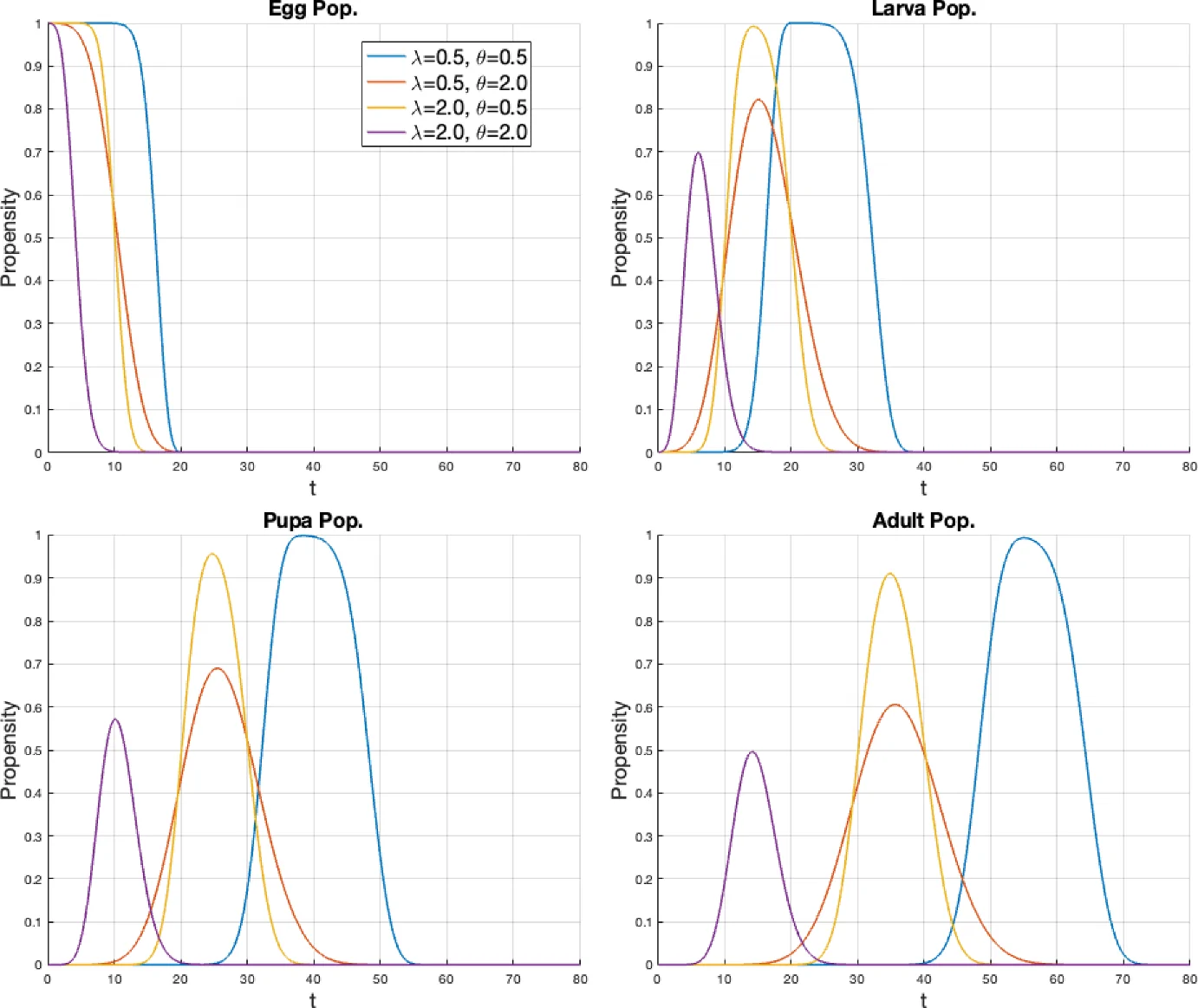
The dynamics, according to the Eq. (3.6), for $x_0=0$, ν=1, $\Delta x_i=20$ for $i\in \{1,2,3,4\}$ and $\lambda ,\theta \in \{0.5,2\}$

The plot of the dynamics are shown in Fig. 2, for constant development rate ν=1, $x_0=0$, $\lambda ,\theta \in \{0.5,2\}$ and each physiological age segment length $\Delta x_i:= x_{i} - x_{i-1}=20$ for $i\in {1,2,3,4}$ (that is assuming that the average development time for each stage is 20 days). It should be noted that λ adjusts the number of steps taken by the Poisson process and θ adjusts the step size of each exponential random variable. Figure 2 provides empirical support for the theoretical findings as:

3.8 $$\begin{aligned} \mathbb {E}[X_t]=x_0 + \bar{\nu }(t) + \lambda t \theta ,\quad Var(X_t) = t\lambda \theta ^2. \end{aligned}$$

As seen by expression (3.8), different pairs of $(\lambda , \theta )$ actually allows to fine tune the model to have any desired pair of expectation and variance. Finally, note that as both the jump number and jump size increase, development is faster.

#### Remark 3.3

Since the area under the Erlang distribution is equal to 1, this property ensures that the total number of individuals, $\sum _{i\in S}N_i(t)$, as defined in expression () is conserved for all t>0, for $S:=\{Eggs,\,Larvae,\,Pupae,\,Adults,\,Dead\}$.

#### Remark 3.4

Because the exponential increments, $G_k$, are all independent and identically distributed, the standard Central Limit Theorem allows us to write $Z_t\sim \mathcal {N}(\lambda t \theta , t\lambda \theta ^2)$ for large *t*. The model developed here is able to approximate the Gaussian framework, thereby inheriting its desirable features, such as its ability to capture the population dynamics observed in real life (see Sperandio et al. 2024; Pasquali and Trivellato 2023; Pasquali et al. 2020, 2022).

### Calibration of the development rate ν(T(t))

In this section, we will discuss how to calibrate the deterministic development rate ν(t) in general, assuming that ν(t) is a function of time via temperature, i.e. $\nu (t)=\nu \left( T(t)\right)$ (see Shi et al. 2016; Briere et al. 1999; Briere and Pracros 1998; Sperandio et al. 2024; Pasquali et al. 2022). The following lemma will be useful in the calibration process.

#### Lemma 3.1

Define the first passage time for this process as:

3.9 $$\begin{aligned} \tau _k := \inf _{t}\{t>0 : X_t\ge x_k\}, \end{aligned}$$

for a fixed $x_k>0$. Then,

$$\begin{aligned} X_{\tau _k}:= x_k + O_k. \end{aligned}$$

where $O_k:=X_{\tau _k}-x_k$, is called the overshoot. $O_k=0$ if the level $x_k$ is reached by the continuous development and $O_k\sim \mathcal {E}xp(\theta )$ if the level $x_k$ is passed by a jump due to the presence of the process $Z_t$ with exponential increments.

#### Proof

When the level $x_k$ is reached by the continuous deterministic drift term $\bar{\nu }(t)$, it is clear that no overshoot can occur.

Now let’s focus on the case when the level $x_k$ is crossed by a jump. Define the jump times as $0<t_{J_1}<t_{J_2}<\dots <t_{J_n}$, where at $t_{J_n}$ the *n*’th jump occurs and $X_{t_{J_n}}$ crosses the level $x_k$. Define the pre-jump level time as $t^\varepsilon _{J_n}:=t_{J_n}-\varepsilon$ for a sufficiently small ε. Using (3.3), one can write down the level reached by the process as:

$$\begin{aligned} X_{t^\varepsilon _{J_n}} = \bar{\nu }(t^\varepsilon _{J_n}) + \sum _{k=0}^{n-1}G_k. \end{aligned}$$

Define the gap between the level $x_k$ and $X_{J_n^\varepsilon }$ as

$$\begin{aligned} R:=x_k - X_{J_n^\varepsilon }. \end{aligned}$$

One can define the overshoot in terms of these objects as:

$$\begin{aligned} O_k = X_{t_{J_n}}-x_k = X_{t^\varepsilon _{J_n}} + \bar{\nu }(t_{J_n}) - \bar{\nu }(t^\varepsilon _{J_n})+G_n-x_k\xrightarrow {\varepsilon \rightarrow 0}G_n - R. \end{aligned}$$

Since we assume that the *n*’th jump $G_n$ causes the level $x_k$ to be crossed, it follows that $G_n > R$, hence:

$$\begin{aligned} \mathbb {P}(O_k> x_k)&=\mathbb {P}(O_k>x_k|G_n> R)\\&\xrightarrow {\varepsilon \rightarrow 0} \mathbb {P}(G_n>x_k + R | G_n>R)\\&=\frac{\mathbb {P}(G_n> x_k + R)}{\mathbb {P}(G_n> R)}\\&=\frac{e^{-x_k/\theta }\mathbb {P}(G_n>R)}{\mathbb {P}(G_n>R)}\\&=e^{-x_k/\theta } \end{aligned}$$

where we also used the independence between $G_n$ and *R*. □

#### Remark 3.5

The choice of i.i.d. exponential increments in the jump process (3.2) is motivated by the memoryless property of the exponential distribution, which is instrumental in proving Lemma 3.1. In particular, this property allows the density of the overshoot to be characterized explicitly.

We write down the identity:

$$\mathbb {E}[X_{\Delta \tau _k}]=\mathbb {E}[\Delta X_{ \tau _k}]=\mathbb {E}[X_{\tau _k} - X_{\tau _{k-1}}] = \mathbb {E}[x_k + O_k - x_{k-1} - O_{k-1}] = \Delta x_k, $$

for $k\in \{1,2,3,4\}$. Because $\mathbb {E}[O_k]=\mathbb {E}[\mathbb {E}[O_k|cross \, by \, jump]]=\mathbb {E}[\theta ]=\theta$ for both *k* and k-1 using Lemma 3.1, they cancel each other.

Because we assumed the deterministic development rate depends explicitly on temperature, for a fixed temperature $T^*\in \mathbb {R}$, $\bar{\nu }(T(t))=\nu (T^*)t$ since $\nu (T^*)$ is just a constant. Taking the expectation of the expression (3.3) at times $\tau _k$ and $\tau _{k-1}$ yields:

3.10 $$\begin{aligned} \Delta x_k = \mathbb {E}[X_{\Delta \tau _k}] = \nu (T^*)\mathbb {E}[\Delta \tau _k] + \lambda \mathbb {E}[ \Delta \tau _k ]\theta \end{aligned}$$

in the above expression, we used the fact that $\mathbb {E}[Z_{\Delta \tau _k}]=\mathbb {E}[\mathbb {E}[Z_{\Delta \tau _k}|\Delta \tau _k]]=\mathbb {E}[\lambda \Delta \tau _k \theta ]$. Once the Eq. (3.10) is rearranged, one obtains:

3.11 $$\begin{aligned} \nu (T^*) = \frac{\Delta x_k}{\mathbb {E}[\Delta \tau _k]} - \lambda \theta . \end{aligned}$$

This shows that, once the physiological age axis has been constructed, so that $\Delta x_k$ is determined and the *average* development times $\mathbb {E}[\Delta \tau _k]$ are known, one can evaluate $(\nu (T^*))$ for a given pair $(\lambda , \theta )$.

### Introducing fecundity and successive generations

**Fig. 3:**
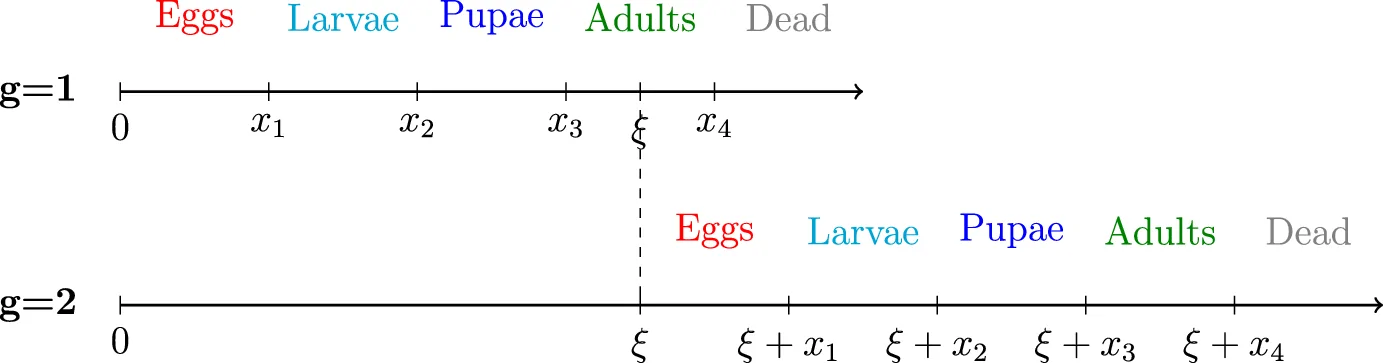
Physiological age axis of the first 2 generations and illustrating the shift between them

Let $X^{(g)}_t$ denote the physiological age of an individual belonging to the *g*’th generation. In this section, we derive the physiological age $X^{(g)}_t$ of an individual starting from the first generation process $X^{(1)}_t$, whose explicit expression is given in (3.6). Assume that an adult of the first generation lays an egg at a fixed physiological age $\xi \in [x_3, x_4)$. Under this assumption, the offspring’s physiological age axis can be thought to be shifted so that the point ξ is the initial condition of the second generation. It is illustrated above in Fig. 3:

Therefore, conditional on the egg being laid at physiological age ξ, the dynamics of the second generation are identical to those of the first generation, differing only by the additive shift ξ, that is the physiological age when the egg is laid.

In practice however, oviposition does not occur at a single physiological age. Adult females may lay eggs throughout their reproductive stage, corresponding to the interval $$[x_3,x_4)$$. To account for this variability, we introduce $$\rho (\xi )$$, the oviposition profile of *C. pomonella*, that is, the likelihood a female lays an egg at physiological age $$\xi \in [x_3,x_4)$$ as also explained in (2.2). Rather than selecting a fixed oviposition age a priori, we average over all possible physiological ages according to ρ. Let

3.12 $$\begin{aligned} \bar{x}:=\int _{x_3}^{x_4}\xi \rho (\xi )d\xi , \end{aligned}$$

be the number which shifts the physiological age axis between generations as illustrated in Fig. 3. according to the oviposition profile of the pest ρ(x). Throughout this work, we assume that the oviposition profile is normalized,

$$\begin{aligned} \int _{x_3}^{x_4}\rho (\xi )d\xi =1 \end{aligned}$$

so that ρ is a probability density function. Under this assumption, the total population is conserved from one generation to the next, consistently with the objectives of phenological models, where the focus is on describing the temporal distribution of individuals across developmental stages rather than changes in population abundance.

Denote by $\mathcal {P}^{(g)}_t(i)$ the propensity of the population of generation $g\in \mathbb {N}$ of stage $i\in \{1,2,3,4\}$ at time t>0. By recursion, the propensities for each stage are given as:

3.13 $$\begin{aligned} \mathcal {P}^{(g)}_t(i) = \mathbb {P}(x_{i-1}\le X^{(g)}_t< x_i) = \mathbb {P}(x_{i-1} + (g-1)\bar{x}\le X^{(1)}_t < x_i+(g-1)\bar{x}) \end{aligned}$$

which depends on the dynamics of the first generation shifted by the term $(g-1)\bar{x}$, and $X^{(1)}_t$ is made explicit in the previous section. In simpler terms, one just replaces $\gamma _i$ in expressions (3.6) with $\gamma _i + (g-1)\bar{x}$.

For the sake of completeness we provide the complete model with other generations below:

3.14 $$\begin{aligned} \begin{aligned} \mathcal {P}^{(g)}_t(Eggs)&= \sum _{n=0}^{\infty } F_{n,\theta }\big (\gamma _{1}+ (g-1)\bar{x}\big ) e^{-\lambda t}\frac{(\lambda t)^n}{n!}\\ \mathcal {P}^{(g)}_t(Larvae)&= \sum _{n=0}^{\infty } \Big ( F_{n,\theta }\big (\gamma _{2}+ (g-1)\bar{x}\big ) - F_{n,\theta }\big (\gamma _{1}+ (g-1)\bar{x}\big )\Big ) e^{-\lambda t}\frac{(\lambda t)^n}{n!}\\ \mathcal {P}^{(g)}_t(Pupae)&= \sum _{n=0}^{\infty } \Big ( F_{n,\theta }\big (\gamma _{3}+ (g-1)\bar{x}\big ) - F_{n,\theta }\big (\gamma _{2}+ (g-1)\bar{x}\big )\Big ) e^{-\lambda t}\frac{(\lambda t)^n}{n!}\\ \mathcal {P}^{(g)}_t(Adults)&= \sum _{n=0}^{\infty } \Big ( F_{n,\theta }\big (\gamma _{4}+ (g-1)\bar{x}\big ) - F_{n,\theta }\big (\gamma _{3}+ (g-1)\bar{x}\big )\Big ) e^{-\lambda t}\frac{(\lambda t)^n}{n!}.\\ \mathcal {P}^{(g)}_t(Dead)&= \sum _{n=0}^{\infty } \Big ( F_{n,\theta }\big (\gamma _{5}+ (g-1)\bar{x}\big ) - F_{n,\theta }\big (\gamma _{4}+ (g-1)\bar{x}\big )\Big ) e^{-\lambda t}\frac{(\lambda t)^n}{n!}, \end{aligned} \end{aligned}$$

where $F_{n,\theta }(x)$ is the Erlang cdf 3.7.

For simplicity, throughout the rest of the paper, let us assume that adults have uniform probability of producing new eggs at every age. Moreover in order to preserve the number of individuals, let us take $\rho (x)=1/(x_4-x_3)=1/\Delta x_4$ for $x\in [x_3, x_4)$. This choice leads to,

$$\begin{aligned} \bar{x}:=\frac{x_4^2 - x_3^2}{2(x_4-x_3)}=\frac{x_3+x_4}{2}, \end{aligned}$$

which is just the midpoint of the adult physiological age interval $$[x_3,x_4)$$. One should emphasize that the choice of the fecundity profile ρ(x) actually affects this number $$\bar{x}$$ via expression (3.12).

**Fig. 4:**
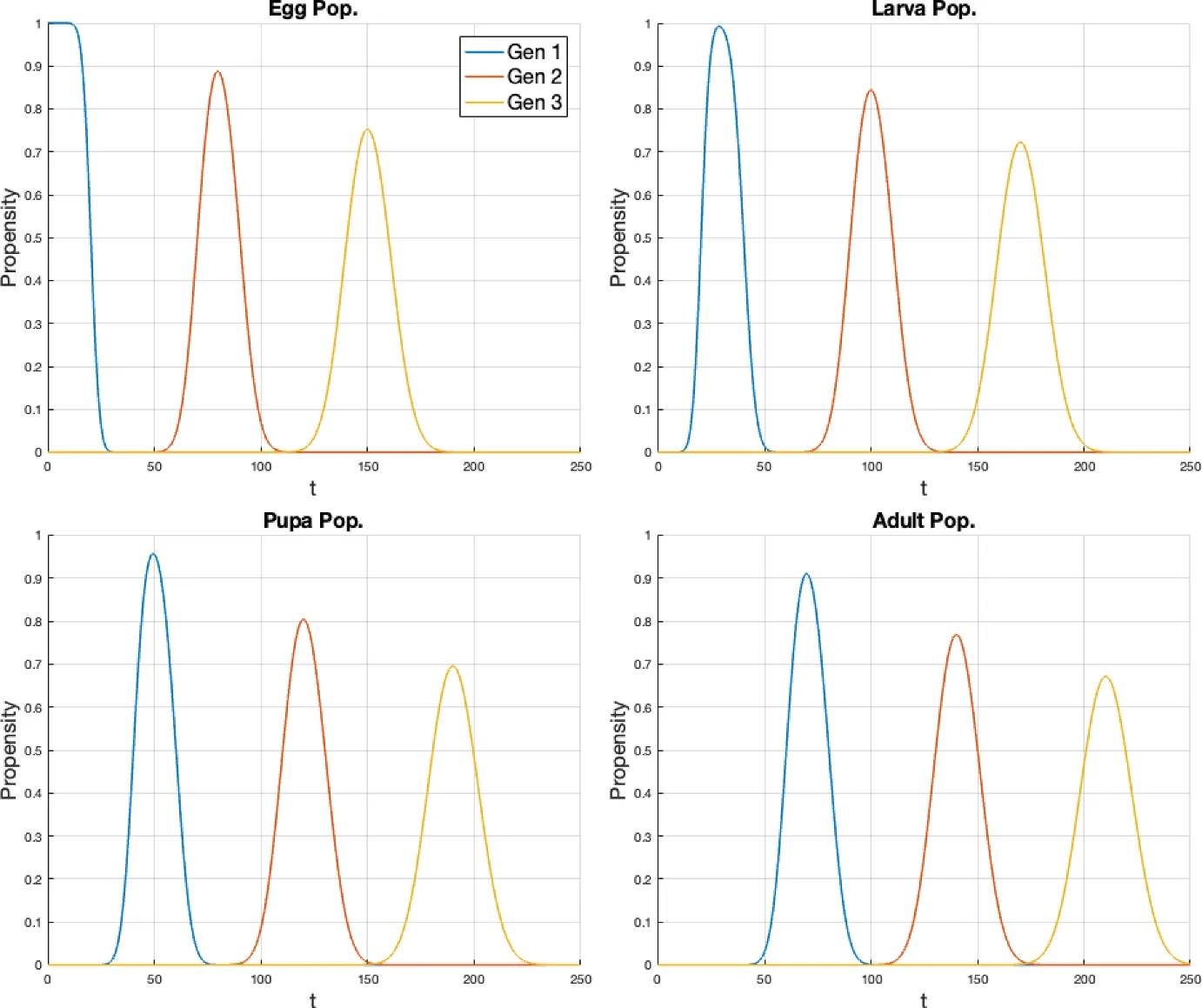
The dynamics with 3 generations, according to the Eq. (3.14), with $x_0=0$, ν=1, $\Delta x_i=40$ for $i\in \{1,2,3,4\}$, $\lambda =\theta =1$ and ρ(x)=1/40

In Fig. 4 are reported the dynamics obtained by considering $x_0=0$, ν=1, $\Delta x_i=40$ for $i\in \{1,2,3,4\}$, $\lambda =\theta =1$ and ρ(x)=1/40.

## Poission model applied on *Cydia pomonella* data

*C. pomonella* (Lepidoptera: Tortricidae), commonly known as codling moth, is one of the most important pests of pome fruits worldwide, representing a major economic concern (Jaffe et al. 2018). It is an obligate carpophagous pest whose larvae feed on fruits, causing direct yield losses and reducing market quality (Guo et al. 2021).

The life cycle of the codling moth is characterized by four developmental stages *i*, namely *i* = 1 for the egg, *i* = 2 for the larva, *i* = 3 for the pupa and *i* = 4 for the adult.

It is a mono- or multivoltine species, with the number of generations varying according to climatic conditions (Balaško et al. 2020). In Italy, it usually completes two generations per year in northern regions, while up to three generations in central and southern areas.

We used four datasets of adult abundances collected in orchards located in Ravenna (year 2022), San Bartolo (years 2021 and 2022), and Piangipane (year 2025), all within the Emilia-Romagna region of Italy. Adult individuals were captured in untreated orchard plots using pheromone traps. At each site, two traps per hectare were installed. Traps were inspected weekly to monitor the presence and abundance of adult individuals, and pheromone dispensers were replaced every eight weeks.

### Physiological age axis construction and development rate calibration

To obtain the development rate functions, we consider the mean development times for immature stages reported in Table 2 of Aghdam et al. (2009b) and the mean longevity for adults reported in Table 1 of Aghdam et al. (2009a) (see Table 1).

**Table 1:** Mean developmental time of the codling moth immature stages (Aghdam et al. 2009b) and mean adult longevity at constant temperatures (Aghdam et al. 2009a)

	Average development durations, $\mathbb {E}[\Delta \tau ]$ (days)			
$\text {Temp}\, (^\circ$C)	Eggs	Larvae	Pupae	Adults
14	18.67	58.34	56.29	25.375
20	9.34	32.38	26.6	12.846
25	4.8	18.77	14.73	10.064
27	4.52	15.44	12.69	7.789
30	4.04	15.62	11.96	7.636
33	4.19	17.22	12.5	6.5

#### Physiological age axis construction

Using Table 1, we compute the proportional duration required for each stage to complete development with respect to the adult longevity (Table 2).

**Table 2:** Proportional development times of each stages with respect to the adult longevity. In the last row an average of proportional durations are taken based on all temperatures

	Proportional development time			
$\text {Temp}\, (^\circ$C)	Eggs/Adults	Larvae/Adults	Pupae/Adults	Adults/Adults
14	0.74	2.30	2.22	1
20	0.73	2.52	2.07	1
25	0.48	1.87	1.46	1
27	0.58	1.98	1.63	1
30	0.53	2.05	1.57	1
33	0.65	2.65	1.92	1
Average	0.62	2.23	1.81	1

Then, using the values reported in Table 2, the physiological age axis can be constructed. The explicit calculation is given by

4.1 $$\begin{aligned} (0, \,0.62, \, 2.85, \,4.66,\,5.66)\times \overline{\Delta x}. \end{aligned}$$

The value $\overline{\Delta x}$ does not affect the proportional development times; rather changes the measuring scale. Consider that moving the scale from a *day* to an *hour*, then one should choose $\overline{\Delta x} = 24$.

##### Remark 4.1

It is important to note that the specific values (0.62, 2.85, 4.66, 5.66) are not intrinsically meaningful. As indicated by the factor $\overline{\Delta x}$ in Eq. (4.1), different choices of $\overline{\Delta x}$ produce different numerical values for the stage boundaries $(x_1, x_2, x_3, x_4)$ while preserving their relative length, which is determined solely by the proportional development times reported in Table 2. Thus, the physiological age axis is constructed by first fixing the relative lengths of the stage intervals according to the developmental times and then selecting an arbitrary value of $\overline{\Delta x}$. Increasing $\overline{\Delta x}$ uniformly stretches the physiological age axis, requiring the stochastic process $X_t$ in Eq. (3.3) to traverse longer intervals. Consequently, the fitted values of ν(t), λ and θ increase accordingly, since the interval lengths enter the calibration through the term $\Delta x_k=(x_k-x_{k-1})\overline{\Delta x}$ in Eq. (3.11). Therefore, the choice of $\overline{\Delta x}$ does not alter the model dynamics; it merely rescales the fitted parameters, corresponding to a different parametrization of the physiological age axis (e.g., hours, days, or any other consistent unit). The calibration procedure presented here exploits this scaling property.

**Fig. 5:**
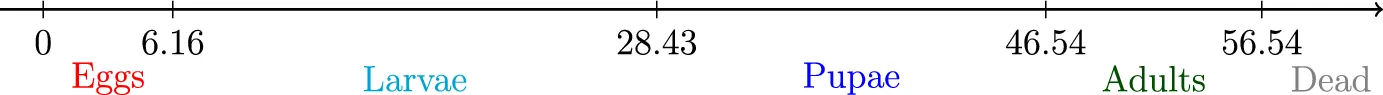
The physiological age axis of the first generation, calculated by (4.1) using $\overline{\Delta x}=10$ with the corresponding development stages labeled

Throughout the remainder of this section, we set $\overline{\Delta x}=10$ for illustrative purposes. The resulting physiological age axis is represented in Fig. 5.

##### Remark 4.2

Since the whole model shares the common development rate ν(t) among all stages, the differences in development times are handled via the different physiological age interval sizes $\Delta x_i$ for $i\in \{1,2,3,4\}$ as shown in Fig. 5.

Since *C. pomonella* overwinters in the larval stage (Sperandio et al. 2024), we assume that the initial population consists of individuals at the onset of the larval stage. Accordingly, the stochastic process in expression (3.3) is initialized at $x_0=6.16$, corresponding to the lower boundary of the larval physiological age interval.

#### Development rate calibration

In this section, we choose a functional form for the deterministic development rate function ν(T(t)), as a function of temperature, and fit its parameters using the methodology described in Sect. 3.2 and the data reported in Table 1. The values shown in Table 1 represent the average number of days required for development at a given stage. For example, larvae at ${14\,}^\circ$C take 58.34 days to complete their development. In other words, Table 1 provides us with the values $\mathbb {E}[\Delta \tau _k]$ for $k\in \{1,2,3,4\}$.

Therefore, in Eq. (3.11) the values of $\Delta x_k$’s are known from the physiological age axis in Fig. 5 and the values $\mathbb {E}[\Delta \tau _k]$’s are known from Table 1. The only unknown remaining is the product $\lambda \theta$, which can be estimated once a form for the development rate function has been chosen. In what follows, we consider the Brière function as the development rate function:

4.2 $$\begin{aligned} \nu (T) = {\left\{ \begin{array}{ll} aT(T-T_{min})(T_{max}-T)^\eta \qquad & T\in [T_{min}, T_{max}]\\ 0 \qquad & otherwise. \end{array}\right. } \end{aligned}$$

The Brière function is commonly preferred in literature (see Shi et al. 2016; Buffoni et al. 2025; Briere et al. 1999) since it captures the linear dependence at low temperatures and manages to stop development after reaching the maximum temperature threshold, where the linear model fails to do so Briere and Pracros (1998) and the symmetry/asymmetry of the function can be adjusted by modifiying the parameter η>0.

**Fig. 6:**
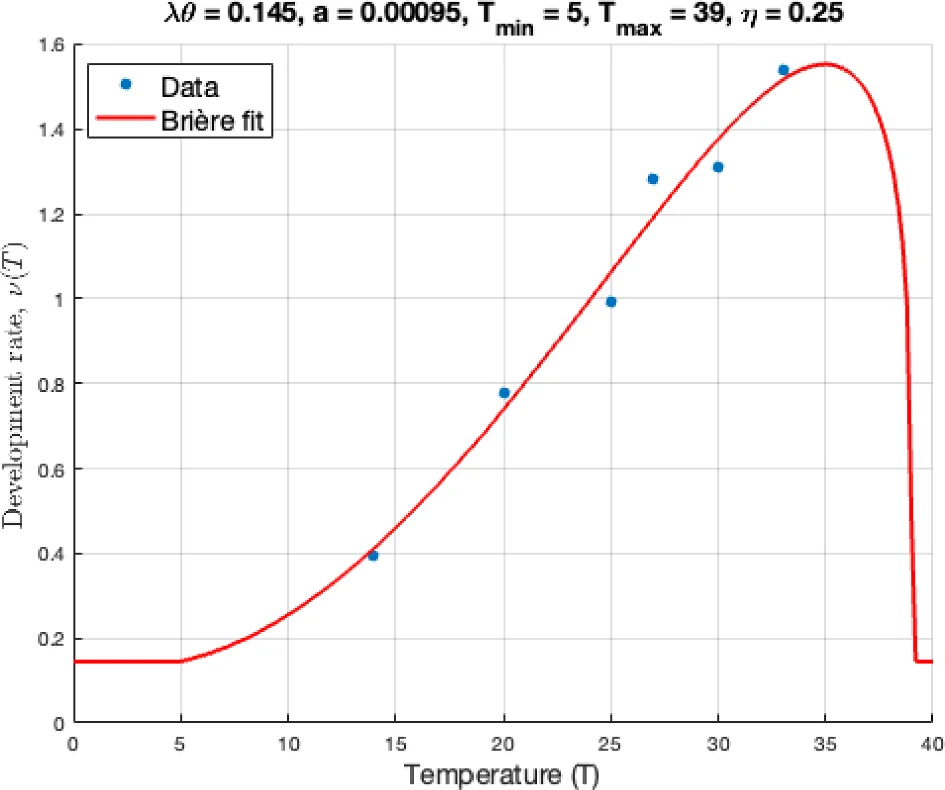
The fit of $\nu (T)+\lambda \theta$ to data points $\Delta x_k/\mathbb {E}[\Delta \tau _k]$ with Brière shape for the development rates. Data points are calculated using the entries of the Table 1 with $\overline{\Delta x}=10$

Using adult development data in Table 1, the parameters $(a, T_{min}, T_{max}, \eta , \lambda \theta )$ have been fitted by least squares estimation to yield a realistic development rate fit for our model (3.3). The resulting fit is given in Fig. 6.

### Dynamics of *C. pomonella*

We are now able to plot the dynamics of *C. pomonella* applying the compound Poisson process (3.14) with the physiological age axes provided in Fig. 5, and the deterministic development rate ν(T(t)) represented in Fig. 6. We start with an initial condition of 100 larvae and use the hourly temperatures recorded by the weather stations located at the sites.

To reflect the dispersion of the data, we chose a pair $(\theta ,\lambda )$ for each site, seeking to obtain the best fit of the collected data. Keeping this in mind, we chose θ=0.4 for Ravenna (2022), which leads us to take λ=0.36 due to the Brière fit in Fig. 6 and θ=2 for San Bartolo (2021, 2022) and Piangipane (2025), which requires us to choose λ=0.07. This means that since the random dimension of the step θ is smaller in the case of Ravenna (2022), the population dynamics will be more deterministic like, as can be seen from the expression (3.8).

**Fig. 7:**
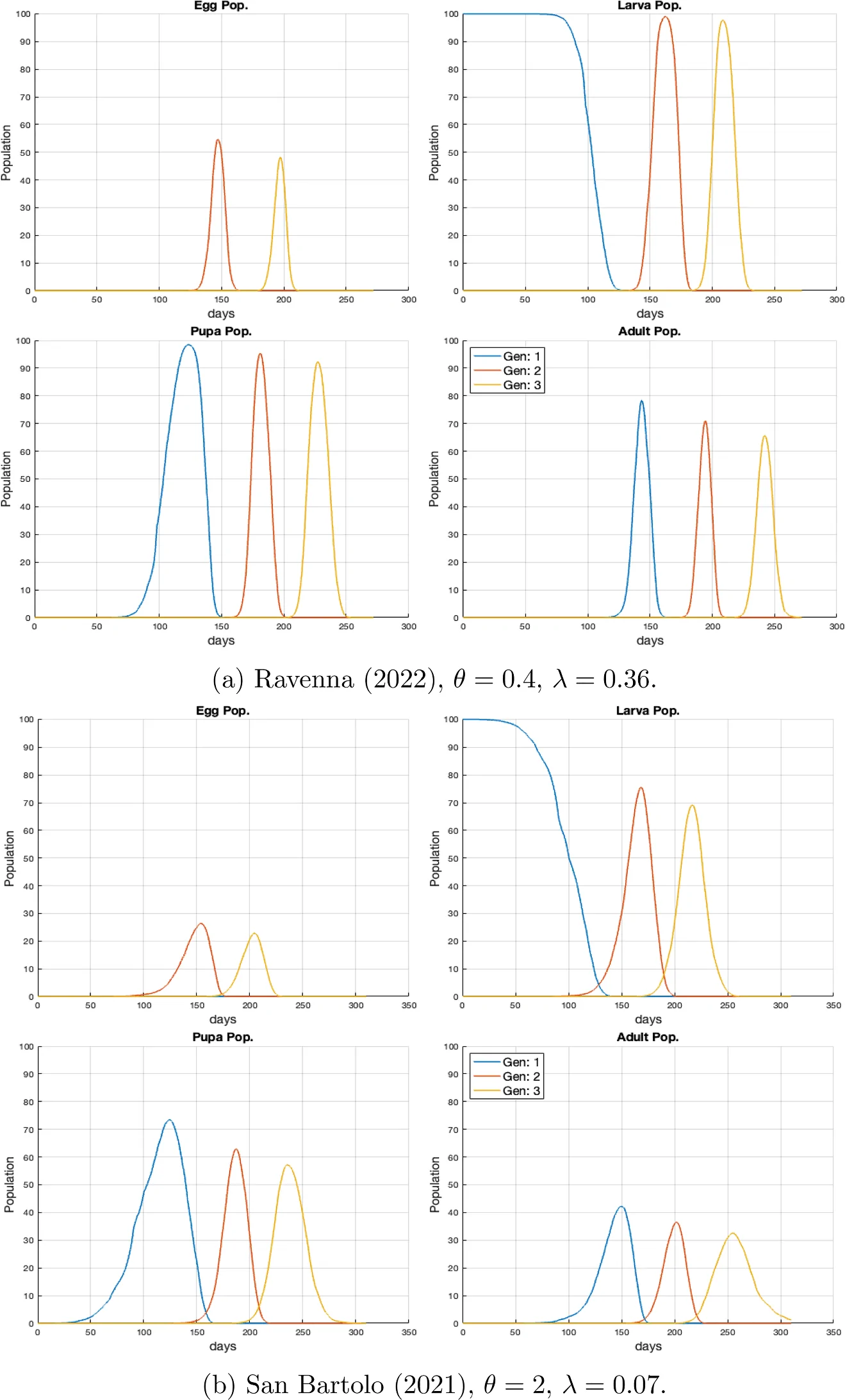
The dynamics of the compound Poisson model (3.14) according to hourly temperatures recorded in indicated fields with $x_0 = 6.16$, 100 initial individuals in larval stage, ν(T) is fitted with the parameters reported in Fig. 6, the values of the pair $(\theta ,\lambda )$ are indicated respectively and $\overline{\Delta x}=10$

When the calculation is performed with the first 500 terms of the infinite series in Eq. (3.14), the Fig. 7 is obtained. Note how the reduced value of θ produces a more deterministic dynamic in Fig. 7a, since there is no overlap between generations, unlike of Fig. 7b. Although not represented here visually, the dynamics for fields of San Bartolo (2022) and Piangipane (2025) also exhibited overlapping generations similar to Fig. 7b.

Since the only data available to validate the model relate to the adult stage, we focus on this stage. The collected abundance data are affected by substantial measurement error, due to the numerous challenges associated with field sampling. Therefore, we employ regularized data, following (Sperandio et al. 2024), and compare cumulative abundances, as is commonly done in the literature. It is calculated by:

$$\begin{aligned} \frac{\text {Area under the peak up to time }t}{\text {Total area under the peak}}. \end{aligned}$$

**Fig. 8:**
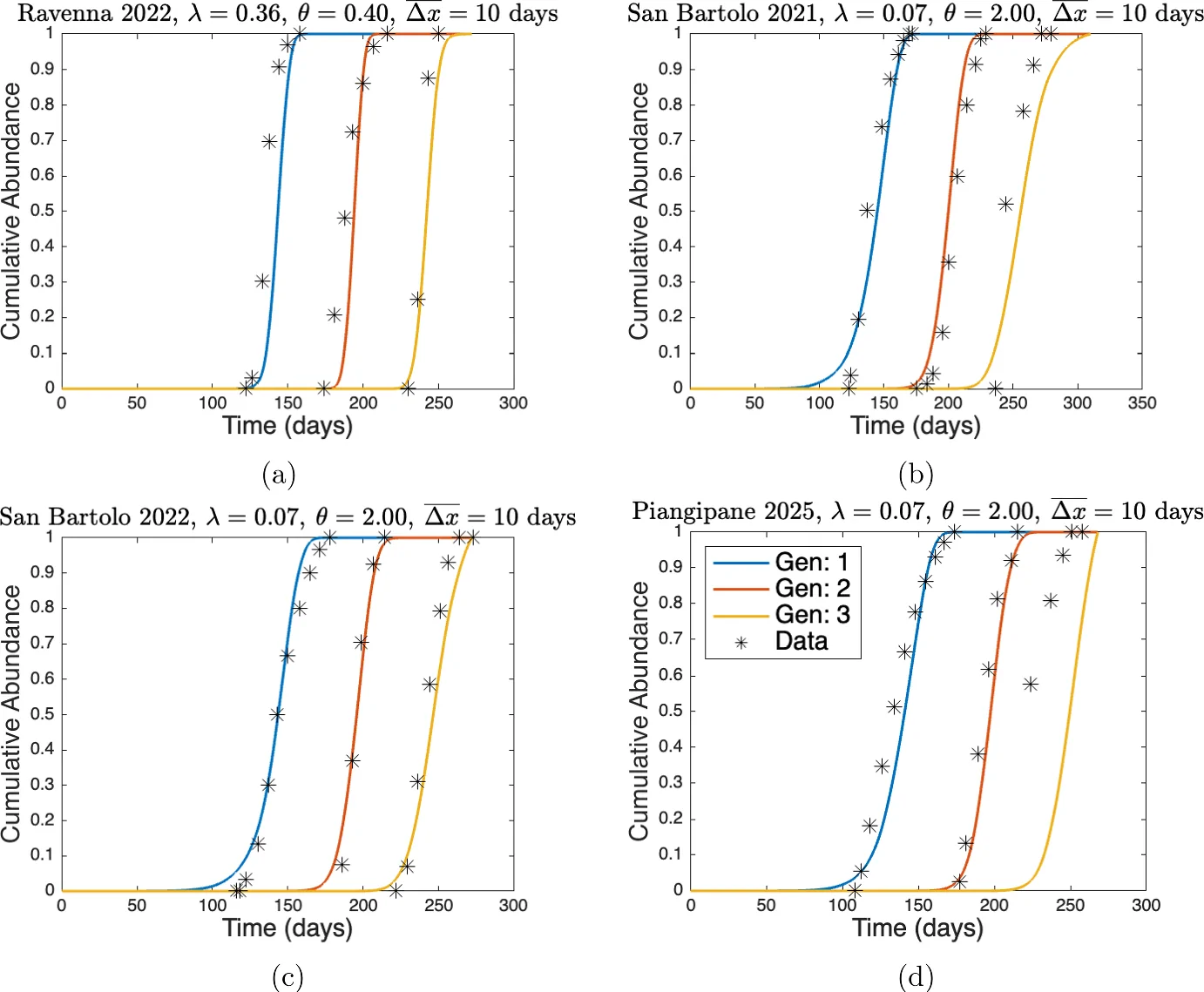
The dynamics of the cumulated abundances for adults obtained using the compound Poisson model in the sites of: Ravenna with parameters λ=0.36 and θ=0.4, San Bartolo and Piangipane with parameters λ=0.07 and θ=2. Asterisks represent the cumulated adult abundances of *C. Pomonella* collected in the field

This approach normalises the amplitude of the peaks and allows us to focus only on the shapes of the peaks at each time point. The value of the cumulated abundances of adults obtained using the compound Poisson process for the sites of Ravenna (2022), S. Bartolo (2021, 2022) and Piangipane (2025) are shown in Fig. 8 and compared with the collected cumulated abundances.

**Fig. 9:**
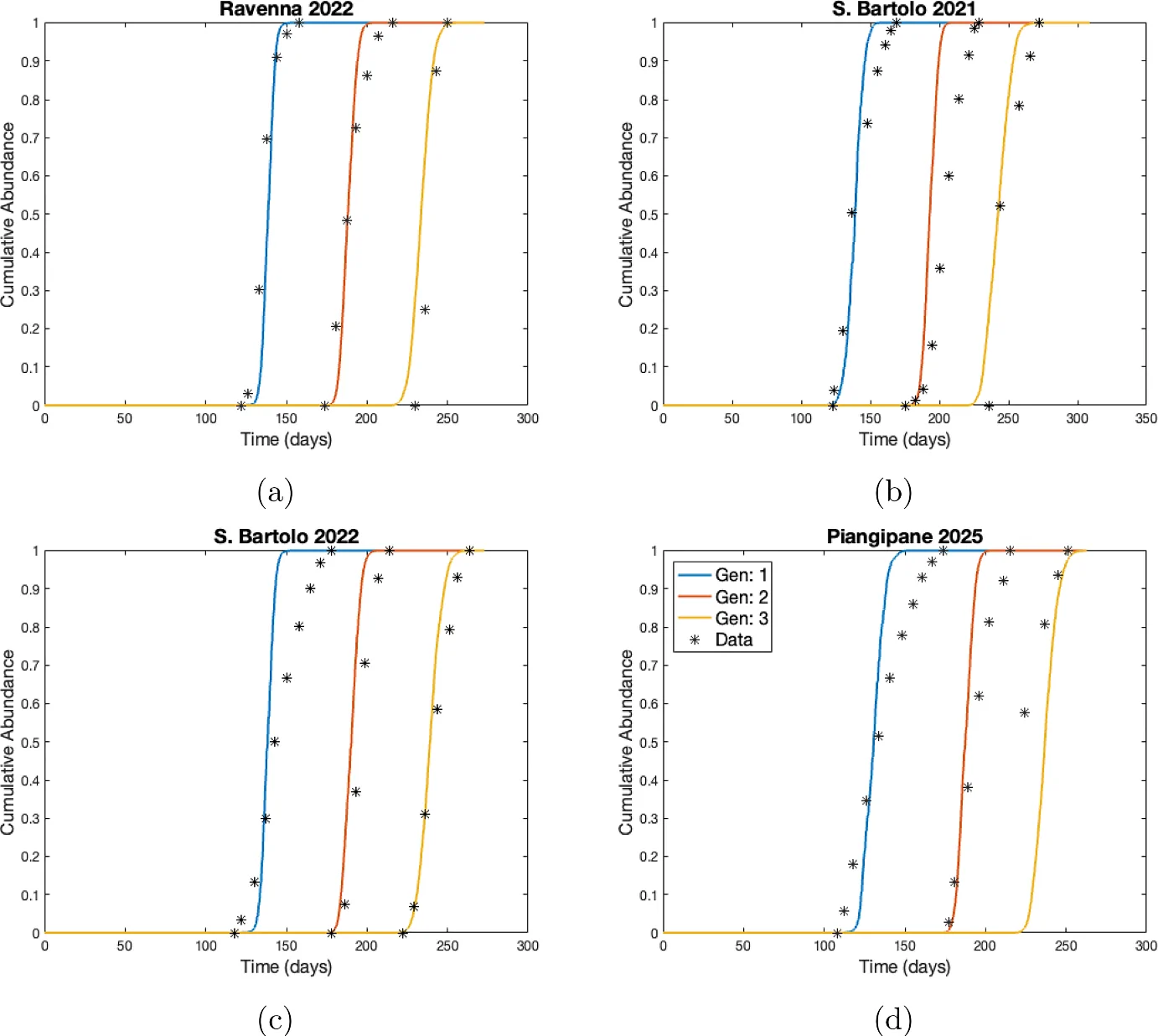
Simulated dynamics of the cumulated abundances for adults obtained using the Gaussian stage structured model (2.4) in the sites of: Ravenna (2022), San Bartolo (2021, 2022) and Piangipane (2025) with $\sigma _i=0.0001\,\forall i\in \{1,2,3,4\}$. Asterisks represent the cumulated adult abundances of *C. Pomonella* collected in the field

For comparison, we simulate the dynamics of *C. pomonella* at the same sites using the Kolmogorov model (2.4).The results of Ravenna and San Bartolo which are previously presented in Sperandio et al. (2024) and Piangipane are shown in Fig. 9. The Kolmogorov model’s fitted Brière development rate parameters are reported in Table 3.

For the Ravenna (2022) dataset, where the observed dynamics appear relatively deterministic, both models reproduce the field data satisfactorily. In contrast, for the other sites, the new model (3.14) provides a noticeably better match during the first two generations, a period in which field data exhibit more regular abundance patterns (with fewer abrupt increases, typically associated with low counts and higher measurement uncertainty).

To quantitatively compare the dynamics obtained with the two models, we computed the mean squared error (MSE) for the four study sites, as shown in Figs. 8 and 9. In all cases, both models exhibit good agreement with the observed data. At S. Bartolo (years 2021 and 2022), the compound Poisson model yields lower MSE values, whereas at Ravenna (year 2022) the Kolmogorov model performs better. For Piangipane (year 2025), the two models produce identical MSE values; however, inspection of the temporal dynamics reveals that the compound Poisson model more accurately captures the first and second generations compared to the Kolmogorov model. In this case, the error is primarily driven by a delayed emergence of the third generation relative to the observed data.

**Table 3:** Parameters *a*, $T_{min}$, $T_{max}$ of the Brière development rate function (4.2) for eggs, larvae and pupae of *C. pomonella* for the Kolmogorov model (2.4) (see Sperandio et al. 2024). Note for all stages, η=0.5

	Eggs	Larvae	Pupae	Adults
*a*	0.0001383	0.0000354	0.000042935	0.00009476
$T_{min}$	9.97	7.57	9.31	9.65
$T_{max}$	38.32	37.03	38.76	35.39

**Table 4:** The mean squared error (MSE) of the two models (2.4) and (3.6) according to how well the adult cumulative abundances fit to the data for the fields in Italy

Mean squared error	Ravenna (2022)	S. Bartolo (2021)	S. Bartolo (2022)	Piangipane (2025)
Kolmogorov Model	0.0244	0.0391	0.0297	0.0393
Compound Poisson Model	0.0557	0.0163	0.0074	0.0393

## Conclusive remarks

In this paper, we introduced an analytic model to describe the dynamics of a pest insect population in which the principal source of stochasticity is represented by a compound Poisson process (3.2), with the increments satisfying $G_k\sim \mathcal {E}xp(\theta )$ with θ>0 as the scale parameter. The proposed approach is conceived as an alternative to the formulations based on partial differential equation systems, such as the von Foerster-type model (2.1) and the Kolmogorov model (2.4), which are widely adopted in the literature for structured population dynamics.

A key advantage of the proposed formulation lies in its analytical tractability. Unlike PDE-based models, which generally require numerical discretization and can be computationally demanding, the compound Poisson framework enables the derivation of explicit expressions for the main dynamical quantities.

The application of the model to the case of the pest *C. pomonella* yielded particularly encouraging results. The compound Poisson model was able to reproduce the observed field dynamics with good accuracy. The comparison with the well-known Kolmogorov model in Figs. 8, 9 and Table 4 highlights an improvement in half of the fields in data fitting. This is particularly evident for sites characterized by overlapping generations. In such cases, the superposition of successive cohorts produces more complex abundance patterns, for which the flexibility of the compound Poisson formulation appears especially advantageous. For the remaining sites, the fit is nonetheless satisfactory, with the additional benefit of a method that does not require discretization of a system of differential equations, but instead allows for the direct derivation of stage abundances.

The flexibility in selecting the parameters λ and θ, which regulate respectively the frequency and the amplitude of the jumps in the compound Poisson process, makes the proposed model highly adaptable to different biological and ecological scenarios. By tuning λ, one can modulate the rate at which stochastic events occur. Similarly, θ controls the expected magnitude of each jump and therefore shapes the intensity of the resulting fluctuations. Their explicit effect on the dynamics is presented in expressions (3.8).

However, it is important to emphasize that, even though one can fit the product $\lambda \cdot \theta$ according to the literature data, the individual choice of the pair $(\lambda ,\theta )$ remains partially arbitrary in practical applications. In principle, if empirical estimates of the variance of the increment process, $Var(X_{\Delta \tau _k})$, were available, expression (3.8) would provide a direct way to calibrate both θ and λ on a statistical basis. In practice, this approach is hindered by the intrinsic difficulty of measuring physiological age quantitatively. Physiological development in insect populations is typically inferred indirectly, for instance through observable life-stage transitions (e.g., egg to larva), rather than through continuous measurements. As a consequence, reliable data on the variance of the underlying physiological-age increments are generally unavailable. This limitation prevents a fully data-driven identification of the pair $(\lambda ,\theta )$ and constrains the calibration procedure to rely on indirect fitting to abundance data.

Despite this constraint, the model retains substantial practical value. The parameters λ and θ can still be estimated by fitting the predicted abundance trajectories to observed time series, thereby ensuring consistency with field data. Moreover, the explicit role played by these parameters in controlling variability offers a clear biological interpretation, which may guide their selection based on ecological knowledge of the species and environmental conditions.

Overall, the proposed phenological model offers a promising and analytically manageable alternative to traditional PDE-based approaches. Future research could extend the framework to include additional biological mechanisms such as diapause, density dependence, or environmental forcing to explore its applicability to other pest species or ecological contexts. Moreover, a natural further development will be the formulation of a demographic compound Poisson model, which would explicitly account for stochastic fluctuations in population size by incorporating random reproduction and mortality events.

## Data Availability

The abundance of the *C. pomonella* adults data for four different fields, used to generate the Figs. 8 and 9 are publicly available on https://doi.org/10.5281/zenodo.19186903.
